# Patterns and correlates of self-reported racial discrimination among Australian Aboriginal and Torres Strait Islander adults, 2008–09: analysis of national survey data

**DOI:** 10.1186/1475-9276-12-47

**Published:** 2013-07-01

**Authors:** Joan Cunningham, Yin C Paradies

**Affiliations:** 1 Menzies School of Health Research, Charles Darwin University, PO Box 41096, Casuarina, Darwin, NT 0811, Australia; 2 Centre for Citizenship and Globalisation, Faculty of arts and Education, Deakin University, 221 Burwood Highway, Burwood, VIC 3125, Australia

**Keywords:** Racism, Discrimination, Aboriginal, Indigenous, Australia

## Abstract

**Background:**

There is now considerable evidence that racism is a pernicious and enduring social problem with a wide range of detrimental outcomes for individuals, communities and societies. Although indigenous people worldwide are subjected to high levels of racism, there is a paucity of population-based, quantitative data about the factors associated with their reporting of racial discrimination, about the settings in which such discrimination takes place, and about the frequency with which it is experienced. Such information is essential in efforts to reduce both exposure to racism among indigenous people and the harms associated with such exposure.

**Methods:**

Weighted data on self-reported racial discrimination from over 7,000 Indigenous Australian adults participating in the 2008–09 National Aboriginal and Torres Strait Islander Survey, a nationally representative survey conducted by the Australian Bureau of Statistics, were analysed by socioeconomic, demographic and cultural factors.

**Results:**

More than one in four respondents (27%) reported experiencing racial discrimination in the past year. Racial discrimination was most commonly reported in public (41% of those reporting any racial discrimination), legal (40%) and work (30%) settings. Among those reporting any racial discrimination, about 40% experienced this discrimination most or all of the time (as opposed to a little or some of the time) in at least one setting. Reporting of racial discrimination peaked in the 35–44 year age group and then declined. Higher reporting of racial discrimination was associated with removal from family, low trust, unemployment, having a university degree, and indicators of cultural identity and participation. Lower reporting of racial discrimination was associated with home ownership, remote residence and having relatively few Indigenous friends.

**Conclusions:**

These data indicate that racial discrimination is commonly experienced across a wide variety of settings, with public, legal and work settings identified as particularly salient. The observed relationships, while not necessarily causal, help to build a detailed picture of self-reported racial discrimination experienced by Indigenous people in contemporary Australia, providing important evidence to inform anti-racism policy.

## Background

Racism is a societal system in which desirable societal resources are differentially allocated to some ethnic/ racial groups above others [1]. Racism can be defined as avoidable and unfair phenomena producing disparities in resources, opportunities or benefits among racial/ethnic groups. It can be expressed through stereotypes (racist beliefs), prejudice (racist emotions) or discrimination (racist behaviours and practices) [2].

Racism can occur at three interrelated conceptual levels that frequently overlap in practice: 1) *internalised racism* - acceptance of attitudes, beliefs or ideologies by members of stigmatised ethnic/ racial groups about the inferiority of one’s own ethnic/racial group (e.g. an Indigenous person believing that Indigenous people are naturally less intelligent than non-Indigenous people); 2) *interpersonal racism* - interactions between people that maintain and reproduce avoidable and unfair inequalities across ethnic/racial groups (e.g. experiencing racial abuse); and 3) *systemic racism* - requirements, conditions, practices, policies or processes that maintain and reproduce avoidable and unfair inequalities across ethnic/racial groups (e.g. Indigenous people experiencing inequitable outcomes in the criminal justice system). This third type of racism is also referred to as institutional racism [2].

Racism is an enduring social problem [3,4], with a wide range of detrimental outcomes for individuals, communities and societies [5]. Although indigenous people worldwide are subjected to high levels of racism [6], there is a paucity of population-based, quantitative data about the factors correlated with their self-reported racial discrimination, about the settings in which such discrimination takes place, or about the frequency with which it is experienced. The New Zealand Health Survey collects data on a limited range of settings (work, housing and medical) as well as ethnically-motivated physical/verbal attack [7], but we are not aware of any other jurisdiction where this occurs. Such information can inform efforts to both reduce exposure to racism and the harms associated with such exposure among indigenous people [8].

In this paper, we examine socio-demographic and socio-cultural correlates of racial discrimination (a mix of interpersonal and systemic racism), including the settings in which it occurs and the frequency with which it is experienced, as reported by Aboriginal and Torres Strait Islander (hereafter referred to as Indigenous) adults in a national Australian survey conducted in 2008–09. Indigenous Australians constitute approximately 2.4% of the Australian population and suffer high rates of unemployment and incarceration, low income, sub-standard housing and a high burden of ill-health and mortality, including a life expectancy 9–12 years less than other Australians [9]. The health and social disadvantage suffered by Indigenous Australians has been associated with both historical and contemporary racism, colonisation and oppression [8]. However, despite the clear impact of racism on Indigenous Australians, detailed data on its prevalence was only collected at a national level in the 2008–09 survey reported on, for the first time, in this paper.

## Methods

Data for Indigenous adults aged 15 years and over were taken from the National Aboriginal and Torres Strait Islander Social Survey (NATSISS), a nationally representative survey of the Australian Indigenous population conducted by the Australian Bureau of Statistics (ABS) in 2008–09 [10,11]. Although this was the third national Indigenous social survey, it was the first to include a detailed set of questions specifically about experiences of racial discrimination.

Extensive details on survey methodology have been published elsewhere [10,11]. Briefly, the survey was conducted using a multi-stage sampling strategy; the first stage involved random selection of either communities or census collection districts (CDs), and subsequent stages involved selection of dwellings and individuals within households [10,11]. The NATSISS sample was designed to provide reliable estimates for Australia as a whole as well as at the State/Territory level. The survey was limited to usual residents of private dwellings and conducted in person by trained ABS interviewers. Data were collected using Computer Assisted Interviews, with paper interview forms available as a back-up in remote areas. There were slight differences in data collection methods used in remote and non-remote areas, with some questions re-phrased or simplified, and some topics deleted [10].

To allow data access to interested researchers, the ABS created a Confidentialised Unit Record File (CURF) for the NATSISS [12]. Although the CURF contains unit records for participants of all ages, this analysis is limited to data from the 7,823 respondents aged 15 years and over. Children less than 15 years have been excluded because they were not asked about their experiences of racial discrimination [11].

### Independent variables

Information was available on a range of socioeconomic, demographic and cultural factors, as shown in Table 1. Information about the age, sex, Indigeneity and relationships of household members was provided by a responsible adult within the household; information about the dwelling, housing tenure, and income of non-participant household members (required to calculate household income) was provided by a household ‘spokesperson’, chosen on the basis of his or her ability to provide accurate information [11]. Information relating to geography (including remoteness classification and area-level disadvantage score) was provided by the ABS based on the CD in which the selected dwelling was located. All other information used in this analysis was provided by the respondent [11].

**Table 1:** **Socio**-**demographic and cultural characteristics of indigenous Australians aged 15 years and over**, **2008**–09*

	**Males**	**Females**	**Total**
	**N = 3,380**	**N = 4,443**	**N = 7,823**
	**% (95% CI)†**	**% (95% CI)†**	**% (95% CI)†**
Age (years)			
15-24	33.4 (33.4-33.4)	30.2 (30.2-30.2)	31.7 (31.7-31.7)
25-34	21.3 (21.3-21.3)	21.5 (21.5-21.5)	21.4 (21.4-21.4)
35-44	18.9 (18.9-18.9)	20.1 (20.1-20.1)	19.5 (19.5-19.5)
45-54	14.1 (14.1-14.1)	14.6 (14.5-14.6)	14.3 (14.3-14.3)
55-64	8.0 (7.9-8.0)	8.3 (8.3-8.3)	8.1 (8.1-8.2)
65-74	3.0 (2.4-3.6)	3.5 (3.1-4.0)	3.3 (2.9-3.6)
75 and over	1.4 (0.1-2.0)	1.8 (1.3-2.3)	1.6 (1.2-2.0)
Married‡	46.8 (44.3-49.2)	42.7 (40.1-45.3)	44.6 (42.5-46.7)
Lives in a remote area§	25.2 (23.9-26.4)	24.7 (23.7-25.7)	24.9 (24.4-25.5)
Level of highest qualification			
University degree	3.7 (2.6-4.9)	5.6 (4.5-6.6)	4.7 (4.0-5.4)
Diploma or certificate	20.9 (18.6-23.2)	20.7 (18.6-22.8)	20.8 (19.1-22.5)
Year 12‖	12.1 (10.2-14.1)	11.9 (10.3-13.5)	12.0 (10.8-13.2)
Year 10 or 11‖	32.5 (29.7-35.3)	33.0 (31.0-35.1)	32.8 (31.0-34.6)
Less than Year 10‖	30.7 (28.1-33.2)	28.8 (26.8-30.8)	29.7 (28.0-31.4)
Labour force status			
Employed	60.5 (57.8-63.3)	43.6 (41.1-46.1)	51.7 (49.5-53.9)
Unemployed	11.7 (9.9-13.5)	8.9 (7.6-10.2)	10.2 (9.1-11.3)
Not in the labour force	27.8 (25.4-30.2)	47.5 (45.1-49.8)	38.1 (36.2-39.9)
Home owner/purchaser**	30.9 (27.7-34.1)	28.2 (25.5-31.0)	29.5 (27.0-32.0)
Equivalised household income quintile††			
1 (lowest)	36.3 (33.0-39.6)	43.2 (40.4-46.1)	39.9 (37.4-42.5)
2	17.7 (15.5-19.9)	16.5 (14.7-18.3)	17.1 (15.6-18.6)
3	12.9 (10.8-14.9)	9.6 (7.8-11.4)	11.2 (9.6-12.7)
4	9.0 (7.5-10.5)	7.4 (5.8-8.9)	8.1 (6.9-9.4)
5 (highest)	4.5 (3.1-6.0)	2.6 (2.0-3.3)	3.5 (2.7-4.4)
Not known or not stated	19.6 (17.2-22.1)	20.7 (18.4-23.0)	20.2 (18.2-22.2)
SEIFA quintile‡‡			
1 (most disadvantaged)	51.0 (45.8-56.1)	55.0 (49.7-60.3)	53.1 (48.2-57.9)
2	18.8 (14.5-23.0)	17.8 (13.7-22.0)	18.3 (14.3-22.2)
3	14.5 (11.1-18.0)	14.6 (11.4-17.9)	14.6 (11.5-17.6)
4	11.6 (8.1-15.0)	8.9 (6.6-11.3)	10.2 (7.5-12.8)
5 (least disadvantaged)	4.2 (2.3-6.2)	3.7 (1.9-5.4)	3.9 (2.2-5.6)
Main language at home is English	86.9 (85.3-88.5)	87.5 (86.3-88.7)	87.2 (86.0-88.5)
Household members all Indigenous	58.3 (55.3-61.3)	64.2 (61.4-67.0)	61.4 (59.0-63.8)
Identifies with a clan, tribal or language group	63.3 (60.4-66.2)	61.0 (58.3-63.6)	62.1 (60.0-64.3)
Recognises homelands	73.3 (70.7-75.9)	70.1 (67.8-72.5)	71.7 (69.8-73.5)
Participated in cultural events, past 12 mos.	58.8 (55.9-61.8)	66.6 (64.1-69.0)	62.9 (60.9-64.9)
Taken away from natural family	8.1 (6.8-9.4)	8.7 (7.3-10.1)	8.4 (7.3-9.5)
How many friends are Indigenous			
Most or all	39.5 (36.7-42.3)	40.3 (37.4-43.2)	39.9 (37.5-42.4)
About half	18.0 (15.8-20.2)	14.3 (12.5-16.2)	16.1 (14.6-17.6)
Few	42.5 (39.6-45.4)	45.4 (42.2-48.5)	44.0 (41.5-46.4)
Level of trust§§			
High	40.1 (37.2-42.9)	33.0 (30.4-35.6)	36.4 (34.3-38.4)
Medium	24.9 (22.1-27.6)	27.1 (24.8-29.5)	26.1 (24.2-28.0)
Low	35.0 (32.1-38.0)	39.9 (37.3-42.4)	37.6 (35.4-39.8)

* Source: Weighted data from the National Aboriginal and Torres Strait Islander Social Survey 2008–09 confidentialised unit record file (CURF) [11,12].

† CI, confidence interval. Proportions are weighted to provide population estimates. Totals are based on those with non-missing data, except for equivalised household income, for which a separate category is shown.

‡ Includes registered marriages and de facto relationships (including same-sex relationships) in which both partners were repored as living in the same household.

§ Classified according to the Australian Standard Geographical Classification remoteness classification (based on the ARIA + index) [11].

‖ Without any post-school qualifications.

** Includes ownership/purchase by any of the dwelling’s occupants (not necessarily the respondent).

††Gross weekly equivalised cash income of household, which takes into account household size and composition, was estimated using the OECD scale [11]. Quintiles were determined based on the household income distribution of the total Australian population.

‡‡SEIFA, Socioeconomic Index for Areas, Index of Relative Disadvantage. SEIFA quintile was based on the 2006 score for the CD of the selected dwelling and is used as a measure of area-level disadvantage [11]. Quintiles were determined based on the total Australian population.

§§Level of trust based on response to the statement “Most people can be trusted”, with responses of ‘disagree’ and ‘strongly disagree’ coded as low trust, ‘neither agree nor disagree’ as medium trust and ‘agree’ and ‘strongly agree’ as high trust.

#### Socio-demographic factors

Respondents who were reported as living in a ‘couple relationship’ (including same sex relationships) were classified as married. Registered marriages and de facto relationships were included when both partners lived in the same household. Respondents who were not reported as being in a couple relationship, as well as those whose partner did not usually live in the same household, were classified as not married [11].

Area of residence was classified according to the Australian Standard Geographical Classification remoteness classification (based on the ARIA index) into major cities, inner regional, outer regional, remote and very remote [11]. However, only two categories (remote and non-remote) were available in the CURF.

Highest educational attainment was calculated by the ABS based on questions about the highest year of school (primary or secondary) completed and the level of any non-school qualifications [11]. For the purposes of analysis, five categories were used: University degree (includes bachelor degree, graduate certificate, graduate diploma, and postgraduate degree); Diploma/Certificate (includes Certificate I-IV or not further defined, Diploma and Advanced Diploma); Year 12 completion (with no non-school qualifications); Year 10 or 11 completion (with no non-school qualifications); and less than Year 10 completion (including never attended) with no non-school qualifications.

Labour force status was classified as employed, unemployed, or not in the labour force [11]. Home ownership was based on whether the home was owned or being purchased by any of its occupants (not necessarily the respondent) [11].

Gross weekly equivalised household income, which takes into account household size and composition, was estimated using the modified Organisation for Economic Co-operation and Development (OECD) scale [11]. Quintiles were determined based on the household income distribution of the total Australian population, as estimated in the 2007–08 Survey of Income and Housing [11]. Household income was not calculated if income information was not available for one or more household members [11].

Area-level disadvantage was based on the 2006 Census-based Socioeconomic Indexes for Areas (SEIFA) Index of Relative Disadvantage score for the CD of the selected dwelling [11]. Quintiles were determined based on all-Australian figures from the 2006 Census.

#### Cultural factors

Respondents were asked several questions about cultural affiliation and participation, including: 1) whether they identified with a tribal group, language group, clan, mission or Aboriginal/Torres Strait Islander regional group; 2) whether they recognised an area as their homelands or traditional country; and 3) whether they had been involved in the past 12 months in cultural events including ceremonies, NAIDOC Week^a^ activities, sports carnivals, art/craft/music/dance festivals or carnivals, Aboriginal or Torres Strait Islander organisations, or funerals/Sorry Business [11]. Respondents were also asked whether they had been taken away^b^ from their natural family.

Respondents were classified as living in an Indigenous-only household if all household members self-identified as Indigenous. The main language spoken at home was classified as English or not English.

Respondents were asked to indicate how many of their friends are Aboriginal or Torres Strait Islander, with possible responses including: all, most, about half, few, none/no friends, and don’t know [11]. For the purposes of analysis, these responses have been grouped into three categories: all/most; about half; and few/none/no friends.

Level of trust was based on response to the statement ‘Most people can be trusted’, with options including: strongly agree, agree, neither agree nor disagree, disagree, and strongly disagree [11]. For the purposes of analysis, these responses have been grouped into three categories: high trust (responded strongly agree or agree); medium trust (neither agree nor disagree); and low trust (disagree/strongly disagree).

### Racial discrimination

The 2008–09 NATSISS included two measures of discrimination, referred to here as racial discrimination and discrimination as a stressor.

#### Racial discrimination

For racial discrimination (the primary measure of interest), respondents were asked whether, in the past 12 months, they had been treated ‘unfairly’ because they were Aboriginal or Torres Strait Islander in any of a specified range of settings and situations. Being treated unfairly was explained as being ‘treated rudely, as if they were inferior or with disrespect; ignored, insulted, harassed, stereotyped or discriminated against; or having unfair assumptions made about them’ [11, p.58]. The specified settings and situations included: 1) applying for work or when at work (WORK); 2) at home, by neighbours or at somebody else’s house (HOME); 3) at school, university, training course or other educational setting (EDUCATION); 4) while doing any sporting, recreational or leisure activities (RECREATION); 5) by the police, security people, lawyers or in a court of law (LEGAL); 6) by doctors, nurses or other staff at hospitals or doctors’ surgeries (MEDICAL); 7) by staff at Government agencies (GOVT); 8) when seeking any other services (e.g. at restaurants, bars, shops, banks, hotels, real estate agencies or in taxis) (SERVICE); 9) by members of the public (e.g. on the street, on public transport, or at shopping centres, parks, libraries, sporting events, concerts, restaurants, pubs or clubs) (PUBLIC); 10) any other situations (OTHER) [11]. Multiple responses were allowed; any settings/situations that were nominated by the respondent were coded as yes, and all other settings were coded as no. The questions and the specified settings were based on the Measure of Indigenous Racism Experiences (MIRE) instrument, which was developed specifically for use in an Australian Indigenous population [13].

Those who responded positively for any of the settings were asked, for each setting, how often they were treated unfairly, with possible responses including: none of the time (0% of the time); a little of the time (1-20%); some of the time (21-50%); most of the time (51-99%); all of the time (100%) [11].

Respondents who did not report being treated unfairly in any of the specified situations/ settings were asked whether they had avoided any of the settings in the past 12 months because of previous experiences of unfair treatment.

#### Discrimination as a stressor

In addition to the specific module on experiences of racial discrimination, respondents were also asked whether any of 24 specified stressors had been a problem for themselves, their family or close friends in the last 12 months. People who reported a stressor were then asked whether it had been a problem for them personally. One of the listed stressors was being ‘treated badly because you are Aboriginal/Torres Strait Islander’ [11].

### Statistical analysis

All analyses were conducted using Stata version 10.0 (StataCorp LP, College Station, TX) via the ABS Remote Access Data Laboratory (RADL). Under the RADL system, submitted statistical code is executed remotely before the output is made available through a password-protected web account. In order to protect the security and confidentiality of the data, there is no direct access to unit record data at any time and there are limits placed on the commands and outputs that are allowed [14].

All analyses used ABS-generated person-weights (or expansion factors) to adjust for disproportionate sampling of some groups. The estimates produced in this manner apply to the population as a whole, and not just the sample being analysed [11,15]. Standard errors and 95% confidence intervals (CI) were calculated using replicate weights (n = 250) produced by the ABS using the Jackknife method [11,15]. These replicate weights allow estimation of standard errors taking into account the complex design and weighting procedures used in the surveys [11,15]. Although Stata version 10 incorporates a suite of procedures to analyse complex survey data, these commands cannot be utilised in the RADL system. Instead, commands from the *svr* module were used [16].

The patterns of exposure to racial discrimination were assessed using weighted proportions. Analysis was conducted for all participants combined, as well as for males and females separately.

The factors associated with reporting of racial discrimination, both overall and in particular settings, were assessed using logistic regression. Analysis was limited to those with non-missing data on all independent variables of interest with the exception of equivalised household income data, for which an ‘unknown’ category was created due to a relatively high proportion of missing values (18.4% of respondents, unweighted). The only other variables with missing data in the ABS CURF were: highest educational attainment (missing for 3.3% unweighted), how many friends are Indigenous (3.2%), whether taken away from family (2.4%), home ownership (0.6%), and whether experienced any of the listed stressors (0.1%); all other variables used in this analysis had complete data.

In logistic regression models with the outcome defined as self-reported discrimination in a particular setting, analysis was limited to those who reported discrimination in that setting and those who did not report discrimination in any setting. Those who reported discrimination in another setting were excluded in order to ensure a consistent comparison group across all models. In some settings (work, education and recreation), models were limited to those aged less than 65 years, due to estimation problems resulting from very low prevalence of self-reported discrimination among older age groups in these settings. The sample size for logistic regression models with any discrimination as the dependent variable was 7,098 (90.7% of all respondents). Models for discrimination in particular settings ranged in size from 4,955 (recreation) to 5,926 (public).

Both crude and adjusted estimates were calculated using logistic regression. Adjustments were made in stages. Model 1 was adjusted for age group, sex and a single socio-demographic or cultural variable. Model 2 was adjusted for age group, sex and all socio-demographic variables of interest. Model 3 was adjusted for age group, sex and all cultural variables of interest. Model 4 was adjusted for age group, sex and all socio-demographic and cultural variables of interest.

Logistic regression was also used to compare those who reported avoiding situations due to past experiences of discrimination with two separate groups: a) those who reported no discrimination in the past 12 months; and b) those who reported discrimination in at least one setting. All comparisons were adjusted for age group and sex.

Logistic regression analysis was also used to examine the factors associated with reporting personal discrimination as a stressor. This analysis was limited to those who reported any racial discrimination, as less than 1% of participants reported personal experiences of discrimination stress without also reporting racial discrimination.

### Ethics approval

This study was approved by both the Aboriginal sub-committee and the main committee of the Human Research Ethics Committee of the Northern Territory Department of Health and Families and Menzies School of Health Research.

## Results

A total of 4,443 females and 3,380 males aged 15 years and over participated in the 2008–09 NATSISS. The socio-demographic and cultural characteristics of participants are shown in Table 1.

As expected, the majority of respondents were young, English-speaking, lacked post-school qualifications, lived in rented housing, had lower levels of household income and lived in relatively disadvantaged areas (Table 1). Males and females were generally similar, but males were more likely than females to be employed and less likely to be classified as not in the labour force. Male participants were also more likely to be married than female participants, and there were differences in household income and SEIFA quintile, with females more likely to be in the most disadvantaged category.

Most participants reported cultural affiliation and participation (Table 1), with females more likely than males to report participating in cultural events in the last year (67% vs. 59% respectively). More than 60% of participants were living in Indigenous-only households, and about 40% indicated that most or all of their friends were Indigenous. A similar proportion of respondents overall were classified as having high (36%) and low (38%) levels of trust, but there were opposite patterns by gender, with males more likely to have high trust and females more likely to have low trust. About 8% of respondents indicated that they had been taken away from their natural family.

### Experiences of racism overall and by setting

More than a quarter of respondents (27%) reported being treated unfairly in the past 12 months because they were Aboriginal and/or Torres Strait Islander (Table 2). Another 4% reported that they had avoided situations due to previous experiences of racial discrimination. Thus nearly one in three respondents (31%) could be classified as having been affected in the past 12 months by racial discrimination. In contrast, about 10% of respondents indicated that they, their family or their friends had experienced racial discrimination as a stressor in the last 12 months, with 6% indicating that they personally had experienced racial discrimination as a stressor.

**Table 2:** **Self**-**reported racial discrimination in the past 12 months for indigenous Australians aged 15 years and over**, **2008**–09*

	**Males**	**Females**	**Total**
	**N = 3,380**	**N = 4,443**	**N = 7,823**
	**% (95% CI)†**	**% (95% CI)†**	**% (95% CI)†**
Reported any discrimination	27.9 (25.2-30.5)	26.8 (24.3-29.2)	27.3 (25.3-29.3)
No discrimination but avoided situations	3.6 (2.7-4.5)	3.9 (3.0-4.9)	3.8 (3.1-4.4)
Affected by discrimination‡	31.4 (28.7-34.2)	30.7 (28.3-33.1)	31.1 (29.0-33.1)
Settings in which discrimination was reported§:			
Public	11.8 (9.6-13.9)	10.7 (9.1-12.4)	11.2 (9.8-12.7)
Legal	14.0 (11.9-16.2)	8.1 (6.8-9.4)	10.9 (9.6-12.3)
Work	9.3 (7.8-10.9)	6.9 (5.7-8.1)	8.1 (7.1-9.0)
Govt	5.7 (4.2-7.3)	4.8 (3.8-5.8)	5.2 (4.3-6.2)
Home	4.3 (3.3-5.4)	5.6 (4.5-6.7)	5.0 (4.2-5.8)
Medical	3.1 (2.3-3.9)	4.8 (3.8-5.9)	4.0 (3.3-4.7)
Service	3.4 (2.4-4.3)	4.3 (3.4-5.2)	3.9 (3.2-4.5)
Education	2.7 (1.8-3.6)	4.6 (3.6-5.6)	3.7 (3.0-4.4)
Recreation	3.8 (2.7-4.9)	2.3 (1.6-3.0)	3.0 (2.3-3.7)
Other	0.3 (0.1-0.4)	0.4 (0.2-0.7)	0.4 (0.2-0.5)
	Mean (95% CI)†	Mean (95% CI)†	Mean (95% CI)†
Number of settings reported‖	2.1 (1.9-2.2)	2.0 (1.9-2.1)	2.0 (1.9-2.1)
Reported discrimination as a stressor	% (95% CI)†	% (95% CI)†	% (95% CI)†
Experienced by self, family or friends	9.8 (8.2-11.3)	9.7 (8.3-11.2)	9.8 (8.6-10.9)
Experienced by self	6.0 (4.7-7.3)	6.1 (4.8-7.4)	6.0 (5.1-7.0)

* Source: Weighted data from the National Aboriginal and Torres Strait Islander Social Survey 2008–09 confidentialised unit record file (CURF) [11,12].

† CI, confidence interval. Figures are weighted to provide population estimates.

‡ Reported discrimination in the past 12 months or avoided situations due to previous experiences of discrimination.

§ PUBLIC = by members of the public (e.g. on the street, on public transport, or at shopping centres, parks, libraries, sporting events, concerts, restaurants, pubs or clubs); LEGAL = by the police, security people, lawyers or in a court of law; WORK = applying for work or when at work; GOVT = by staff at Government agencies; HOME = at home, by neighbours or at somebody else’s house; MEDICAL = by doctors, nurses or other staff at hospitals or doctors’ surgeries; SERVICE = when seeking any other services (e.g. at restaurants, bars, shops, banks, hotels, real estate agencies or in taxis); EDUCATION = at school, university, training course or other educational setting; RECREATION = while doing any sporting, recreational or leisure activities; OTHER = any other situations. Respondents could report discrimination in more than one setting [11].

‖ Among those reporting any discrimination.

Among females, racial discrimination was most commonly reported in public (11%), legal (8%) and work (7%) settings (Table 2). Males experienced racial discrimination in these same settings, with legal (14%) the most commonly reported setting, followed by public (12%) and work (9%) settings. Among those reporting any racial discrimination (i.e., those who reported racial discrimination in at least one setting), about four in ten reported this experience in public (41%) or legal (40%) settings, and about three in ten in a work setting (30%) (Table 3). Among those reporting any racial discrimination, the mean number of settings in which discrimination was reported was two (Table 2).

**Table 3:** **Self**-**reported racial discrimination in the past 12 months by age group and setting**, **indigenous Australian adults aged 15 years and over**, **2008**–09*

	**Any discrimination**	**Discrimination as a stressor (self)**	**Discrimination by setting†**									**No. of settings‡**
			**Public**	**Legal**	**Work**	**Govt**	**Home**	**Medical**	**Service**	**Educ.**	**Recr.**	
**Age (years)**	**% (95% CI)§**	**% (95% CI)§**	**%**	**%**	**%**	**%**	**%**	**%**	**%**	**%**	**%**	**Mean**
15-24	25.5 (22.2-28.9)	5.0 (3.5-6.5)	10.4	10.7	6.8	4.1	4.8	2.5	1.6	5.9	3.4	2.0
25-34	30.5 (27.1-33.9)	6.3 (4.8-7.8)	14.0	11.9	8.0	4.9	6.2	4.2	6.1	2.2	4.0	2.0
35-44	32.6 (29.1-36.2)	8.1 (5.8-10.4)	13.2	13.8	12.1	6.8	6.5	5.2	6.4	3.7	3.5	2.2
45-54	27.3 (23.4-31.3)	7.9 (4.9-10.8)	10.7	11.0	8.6	7.3	3.9	6.1	3.8	2.3	1.9	2.0
55-64	23.5 (18.2-28.7)	4.0 (2.0-6.1)	8.2	7.6	6.3	5.1	3.1	4.5	3.0	3.2	1.0	1.8
65-74	10.5 (5.5-15.4)	1.9 (0.1-3.8)	3.6	2.3	1.7	2.9	1.3	1.0	0.3	0.1	1.3	1.4
75+	7.3 (1.2-13.3)	0.3 (0.0-0.8)	2.7	0.6	1.4	1.9	1.3	0.3	0.6	0.1	0.0	1.3
All adults	27.3 (25.3-29.3)	6.0 (5.1-7.0)	11.2	10.9	8.1	5.2	5.0	4.0	3.9	3.7	3.0	2.0
			%	%	%	%	%	%	%	%	%	Mean
% who reported discrimination in this setting‡			41.2	40.0	29.5	19.2	18.3	14.7	14.2	13.5	11.0	2.0

* Source: Weighted data from the National Aboriginal and Torres Strait Islander Social Survey 2008–09 confidentialised unit record file (CURF) [11,12]. Figures are weighted to provide population estimates.

† PUBLIC = by members of the public (e.g. on the street, on public transport, or at shopping centres, parks, libraries, sporting events, concerts, restaurants, pubs or clubs); LEGAL = by the police, security people, lawyers or in a court of law; WORK = applying for work or when at work; GOVT = by staff at Government agencies; HOME = at home, by neighbours or at somebody else’s house; MEDICAL = by doctors, nurses or other staff at hospitals or doctors’ surgeries; SERVICE = when seeking any other services (e.g. at restaurants, bars, shops, banks, hotels, real estate agencies or in taxis); EDUC. = at school, university, training course or other educational setting; RECR. = while doing any sporting, recreational or leisure activities [11].

‡ Among those reporting any discrimination.

Both racial discrimination and discrimination as a stressor increased with age, peaking among those aged 35–44 years and then declining, with markedly lower reporting among those aged 65 years and over (Table 3). Older respondents were less likely to report racial discrimination and did so in fewer settings on average (Table 3).

In all age groups from 15 to 64 years, the three settings in which racial discrimination was most commonly reported were legal, public and work (Table 3). For those aged 65 years and over, government was the second most common setting after public settings.

Public, legal and work settings were the top three settings in a broad range of subgroups, although the order varied by subgroup (data not shown). Most notably, although work was generally the third most common setting, it was first among those who were unemployed (presumably in relation to applying for work rather than at work) and second among homeowners and those with a university degree.

### High frequency exposure

More than one in ten respondents (11%) reported experiencing racial discrimination most or all of the time in at least one setting (Table 4), with similar proportions for males (11.0%) and females (10.5%). Among those who reported any racial discrimination, about 40% reported that it occurred most or all of the time in at least one setting, with about 6% reporting such high frequency in three or more settings (Table 4). The proportion of participants who reported experiencing discrimination most or all of the time varied according to specific settings, ranging from 48% of those reporting any discrimination in a legal setting to 24% of those reporting any discrimination in a public or recreation setting (Table 5). Reporting was generally similar for males and females, with the exception of legal, medical and public settings. Over half of males reporting racial discrimination in legal settings (51%) – about 1 in 14 males overall (7%) – reported this experience most or all of the time (Table 5). Among females, 44% of those reporting discrimination in legal settings – 4% of females overall – reported this experience most or all of the time. Among those reporting discrimination in medical settings, males were more likely than females to report that it occurred most or all of the time (45% versus 25%, respectively). Conversely, among those reporting discrimination in public settings, females were more likely than males to report that it occurred most or all of the time (31% versus 17%, respectively).

**Table 4:** Number of settings in which respondents reported experiencing racial discrimination most or all of the time*

	**All participants**	**Those reporting any discrimination**
**Number of settings**	**% (95% CI)**	**% (95% CI)**
0	89.2 (87.8-90.6)	60.5 (56.5-64.5)
1	6.5 (5.5-7.4)	23.7 (20.6-26.7)
2	2.7 (1.9-3.4)	9.7 (7.2-12.2)
3	1.0 (0.6-1.4)	3.8 (2.3-5.2)
4	0.4 (0.2-0.5)	1.3 (0.7-1.9)
5 or more	0.3 (0.1-0.5)	1.0 (0.3-1.7)

* Source: Weighted data from the National Aboriginal and Torres Strait Islander Social Survey 2008–09 confidentialised unit record file (CURF) [11,12].

**Table 5:** Proportion of respondents reporting racial discrimination most or all of the time in at least one setting*

	**All participants**			**Those reporting discrimination in this setting**		
**Setting**	**Males**	**Females**	**Total**	**Males**	**Females**	**Total**
	**% (95% CI)**	**% (95% CI)**	**% (95% CI)**	**% (95% CI)**	**% (95% CI)**	**% (95% CI)**
Any	11.0 (9.1-13.0)	10.5 (8.8-12.3)	**10.8 (9.4-12.2)**	39.6 (34.2-45.0)	39.3 (34.4-44.2)	**39.5 (35.5-43.5)**
Legal	7.2 (5.5-8.9)	3.5 (2.7-4.4)	**5.3 (4.3-6.3)**	51.4 (43.7-59.1)	43.8 (35.7-51.9)	**48.5 (42.3-54.6)**
Public	2.0 (1.1-2.8)	3.3 (2.1-4.5)	**2.7 (1.8-3.6)**	16.8 (10.1-23.5)	30.7 (21.8-39.6)	**23.7 (17.1-30.3)**
Work	2.6 (1.8-3.3)	2.0 (1.3-2.6)	**2.2 (1.8-2.7)**	27.4 (20.0-34.8)	28.4 (20.4-36.4)	**27.9 (22.2-33.5)**
Govt	2.5 (1.3-3.7)	1.9 (1.3-2.5)	**2.2 (1.5-2.9)**	44.3 (30.0-58.6)	39.0 (28.6-49.5)	**41.8 (32.6-50.9)**
Home	1.0 (0.5-1.4)	1.7 (1.1-2.3)	**1.3 (0.9-1.7)**	21.6 (12.0-31.3)	30.6 (21.8-39.3)	**26.9 (19.8-33.9)**
Medical	1.4 (0.7-2.1)	1.2 (0.7-1.7)	**1.3 (0.9-1.7)**	45.2 (30.5-60.0)	25.0 (15.5-34.4)	**32.4 (24.0-40.8)**
Education	0.8 (0.1-1.3)	1.4 (0.8-2.0)	**1.1 (0.7-1.5)**	27.0 (8.1-45.8)	31.0 (20.2-41.8)	**29.6 (20.2-39.0)**
Service	0.7 (0.3-1.1)	1.2 (0.8-1.7)	**1.0 (0.7-1.3)**	20.8 (8.7-32.9)	28.8 (19.1-38.5)	**25.5 (18.0-32.9)**
Recreation	1.1 (0.5-1.7)	0.4 (0.2-0.6)	**0.7 (0.4-1.0)**	28.3 (14.4-42.2)	18.0 (8.5-27.5)	**24.2 (15.2-33.2)**

* Source: Weighted data from the National Aboriginal and Torres Strait Islander Social Survey 2008–09 confidentialised unit record file (CURF) [11,12].

### Factors associated with reporting of racism experiences

Several cultural and socio-demographic factors were significantly associated with self-reported racial discrimination in multiple logistic regression models, both overall (Table 6) and in specific settings (Additional file 1, Additional file 2 and Additional file 3). Home ownership, reporting relatively few Indigenous friends, and remote area residence were all associated with lower reporting of racial discrimination in the last year, while being taken away from family, low levels of trust, unemployment, having a university degree, and markers of cultural affiliation/participation were associated with a greater likelihood of self-reported racial discrimination. Similar results were observed for reported racial discrimination in public, legal and work settings (Additional file 1, Additional file 2 and Additional file 3, respectively), and in other settings (data not shown).

**Table 6:** **Relative odds of any self**-**reported racial discrimination in the last 12 months**, **indigenous Australian adults aged 15 years and over**, **2008**–**09**†,‡

	**Model 1§**	**Model 2§**	**Model 3§**	**Model 4§**
	**OR (95% CI)**	**OR (95% CI)**	**OR (95% CI)**	**OR (95% CI)**
Married	0.7 (0.6-0.9)**	0.8 (0.7-1.0)*	---	0.9 (0.7-1.1)
Remote area residence	0.9 (0.7-1.1)	0.8 (0.7-1.0)	---	0.6 (0.4-0.7)***
Highest qualification				
University degree	1.9 (1.2-2.8)**	2.5 (1.6-3.9)***	---	2.0 (1.3-3.1)**
Diploma/certificate	1.2 (0.9-1.6)	1.4 (1.1-1.8)*	---	1.3 (1.0-1.8)
Year 12 only	0.8 (0.6-1.1)	0.9 (0.7-1.2)	---	0.9 (0.6-1.2)
Year 10/11 only	1.0	1.0	---	1.0
<Year 10 only	1.1 (0.9-1.4)	1.0 (0.8-1.3)	---	1.0 (0.8-1.4)
Labour force status				
Employed	1.0	1.0	---	1.0
Unemployed	2.1 (1.6-2.8)***	2.0 (1.5-2.6)***	---	1.8 (1.3-2.5)***
Not in labour force	1.3 (1.1-1.6)*	1.3 (1.0-1.6)*	---	1.2 (1.0-1.5)
Home owned or being purchased	0.5 (0.4-0.6)***	0.5 (0.4-0.6)***	---	0.6 (0.5-0.8)**
Equivalised household income quintile				
1 (lowest)	1.0	1.0	---	1.0
2	0.7 (0.6-0.9)*	0.9 (0.7-1.1)	---	0.9 (0.7-1.2)
3	0.9 (0.7-1.2)	1.2 (0.9-1.6)	---	1.4 (0.9-1.9)
4	0.6 (0.4-0.8)**	0.8 (0.6-1.2)	---	0.9 (0.6-1.3)
5 (highest)	1.2 (0.6-2.2)	1.5 (0.8-3.1)	---	1.7 (0.8-3.5)
Not known/Not stated	0.9 (0.7-1.1)	1.0 (0.8-1.3)	---	1.0 (0.8-1.3)
SEIFA quintile				
1 (most disadvantaged)	1.0	1.0	---	1.0
2	1.2 (0.9-1.6)	1.2 (0.9-1.6)	---	1.4 (1.0-1.9)*
3	0.6 (0.5-0.9)**	0.7 (0.5-0.9)*	---	0.7 (0.5-1.0)
4	1.0 (0.7-1.5)	1.1 (0.8-1.7)	---	1.3 (0.9-2.0)
5	1.0 (0.4-2.1)	1.1 (0.5-2.4)	---	1.3 (0.5-3.1)
Main language not English	1.0 (0.7-1.2)	---	0.5 (0.4-0.7)***	0.8 (0.6-1.1)
Household members all Indigenous	1.8 (1.5-2.3)***	---	1.4 (1.1-1.7)*	1.2 (0.9-1.6)
Identifies with clan, tribal, language group	2.5 (2.0-3.0)***	---	1.6 (1.3-2.1)***	1.6 (1.3-2.1)***
Identifies homelands	2.6 (2.0-3.3)***	---	1.6 (1.2-2.1)**	1.6 (1..2-2.1)**
Participated in cultural events, past 12 months	2.2 (1.8-2.7)***	---	1.5 (1.2-1.8)**	1.5 (1.2-1.9)**
Taken away from natural family	2.6 (2.0-3.3)***	---	2.2 (1.7-3.0)***	2.1 (1.5-2.8)***
% friends who are Indigenous				
Most or all	1.0	---	1.0	1.0
About half	0.9 (0.7-1.2)	---	0.9 (0.7-1.2)	0.9 (0.6-1.1)
Few	0.4 (0.3-0.5)***	---	0.6 (0.4-0.7)***	0.5 (0.4-0.6)***
Level of trust				
High	1.0	---	1.0	1.0
Medium	1.4 (1.1-1.7)**	---	1.3 (1.0-1.7)*	1.3 (1.0-1.6)
Low	2.0 (1.6-2.4)***	---	2.0 (1.6-2.4)***	2.0 (1.6-2.4)***

† Source: Weighted data from the National Aboriginal and Torres Strait Islander Social Survey 2008–09 confidentialised unit record file (CURF) [11,12].

‡ Includes those with complete data on all variables of interest (N = 7,098).

§ Model 1: Adjusted for age group and sex and the variable shown.

Model 2: Adjusted for age group, sex and the socio-demographic variables listed.

Model 3: Adjusted for age group, sex, and the cultural variables listed.

Model 4: Adjusted for age group, sex, and the socio-demographic and cultural variables listed.

* p < 0.05; ** p < 0.01; *** p < 0.001.

### Avoiding settings due to past unfair treatment

Among those who did not report any racial discrimination in the last 12 months, the factors associated with reporting avoiding settings due to past unfair treatment were generally very similar, in both direction and magnitude, to those associated with reporting racial discrimination as described above, with one main exception. Compared with those who reported no experiences of racial discrimination, having a university degree was associated with higher reporting of racial discrimination (odds ratio (OR) = 1.9, 95% confidence interval (CI) = 1.2-2.8), but lower reporting of avoiding situations (OR = 0.7, 95% CI 0.4-1.5), although the latter association was not statistically significant and the two sets of confidence intervals overlap.

Among those who were affected by racial discrimination, either by experiencing it in at least one setting or avoiding situations, the relative odds of reporting avoiding situations (versus reporting any racial discrimination) were higher for those in remote areas (age-and sex-adjusted OR = 1.5, 95% CI = 1.0-2.1) and lower for those with a university degree (OR = 0.4, 95% CI = 0.2-0.9), low levels of trust (OR = 0.6, 95% CI = 0.4-0.9) and those who participated in cultural activities in the past 12 months (OR = 0.7, 95% CI = 0.5-1.0).

Those who did not report racial discrimination but who reported avoiding situations due to past unfair treatment were most likely to report avoiding situations in work, public and legal settings. These same three settings were the most commonly avoided for both males and females.

### Discrimination as a stressor

Participants were over four times more likely to respond positively to the specific question on racial discrimination in the past 12 months than they were to report personally experiencing discrimination as part of a diverse list of stressors: 27% (95% CI 25-29%) versus 6% (95% CI 5-7%), respectively. This disparity was evident for both males and females and was reasonably consistent across age groups. A small group of participants (<1%) reported personally experiencing discrimination as a stressor without also reporting racial discrimination in the past 12 months.

Among those who reported any racial discrimination, several factors were significantly associated with also reporting personal discrimination as a stressor. Removal from family (OR = 2.5, 95% CI 1.6-4.0), having a university degree (OR = 2.5, 95% CI 1.3-4.9), home ownership (OR = 1.9, 95% CI 1.2-3.1), identification with a clan (OR = 1.9, 95% CI 1.2-3.0) and low trust (OR = 1.7, 95% CI 1.1-2.7) were all associated with greater reporting of personal discrimination as a stressor, while remote residence was associated with lower reporting (OR = 0.4, 95% CI 0.3-0.6). Settings were also associated with reporting of personal discrimination as a stressor, with the highest odds ratios observed for discrimination in home settings (OR = 3.3, 95% CI 2.2-5.0) and public settings (OR = 2.8 95% CI 1.9-4.1). The reporting of personal discrimination as a stressor increased with the number of settings in which racial discrimination was reported, as well as with the frequency of exposure. Compared with those reporting racial discrimination in only one setting, those who reported experiencing discrimination in two settings had a relative odds of 1.9 (95% CI 1.1-3.2), rising to 5.6 (95% CI 3.8-8.2) for those reporting three or more settings. For those reporting discrimination most or all of the time in at least one setting, the relative odds of reporting personal discrimination as a stressor was 2.0 (95% CI 1.4-2.9). After adjusting for age, sex and number of settings, remote residence (OR = 0.4, 95% CI 0.3-0.6), home ownership (OR = 1.9, 95% CI 1.1-3.2) and removal from family (OR = 2.6, 95% CI 1.5-4.5) remained significantly associated with reporting personal discrimination as a stressor.

## Discussion

These data from a nationally representative sample of Indigenous Australians indicate that racial discrimination is commonly experienced across a wide variety of settings, with public, legal and work settings identified as particularly salient. Several variables were significantly associated with reporting racial discrimination, ranging from basic demographic measures, such as age and place of residence, to traditional socioeconomic indicators, such as education, housing and employment, to markers of cultural affiliation and participation and levels of trust. These relationships, while not necessarily causal, help to build a detailed picture of racial discrimination experienced by Indigenous people in contemporary Australia.

Almost one in three respondents reported being affected by racial discrimination in the year prior to the survey, either directly (27%) or through avoiding situations due to past experiences (4%). More than one in ten respondents indicated that they had experienced racial discrimination most or all of the time in at least one setting. These figures represent a substantial exposure at the population level, with potentially important negative consequences for health and wellbeing [17].

Across a number of previous studies of various sizes and in various locations in Australia, the prevalence of self-reported racial discrimination among Indigenous Australians has ranged from about 16% to about 97%. This variation is due to factors common to survey research, including the nature and number of questions asked about racism, respondent characteristics and the way in which the sample was selected. For example, a 2003 survey of residents in a rural town found that 40% of Aboriginal respondents reported being physically or emotionally upset in the past 4 weeks as a result of treatment based on their race [18]. In a 2006–08 study of youth who had been born at a single hospital 16–20 years earlier, 32% of 345 respondents reported experiencing a ‘fair bit’ or ‘lots’ of racial discrimination, with no time frame specified [19]. Of the 1,073 children aged between 12 and 17 years in the 2001–2 Western Australian Aboriginal Child Health Survey, about 22% reported being treated badly or refused service in the past six months due to being Aboriginal [20]. Of about 9,400 Indigenous respondents in the 2002–3 NATSISS, 18% reported experiencing discrimination as a stressor in the past twelve months [21]. More recently, about 97% of 755 Aboriginal Australians in convenience samples in two rural and two metropolitan areas of Victoria reported racial discrimination in the 2010–11 Localities Embracing and Accepting Diversity (LEAD) surveys [22]. Of these existing data, only the 2002–3 NATSISS also included a nationally representative sample, with only a single item used in this survey to assess experiences of racial discrimination.

Less than a quarter of those who reported racial discrimination also indicated that they had personally experienced racism as a stressor, with stress being about three times more common for those reporting discrimination in home and public settings. Exploratory research in the U.S. suggests that major experiences of racial discrimination such as being denied a job are experienced as stressful [23], but this is not necessarily the case for more common everyday experiences. Evidence from Aboriginal respondents in the LEAD study supports this, with racial discrimination involving property damage, in seeking housing as well as being left out or avoided more strongly associated with psychological distress than other types of discrimination [22]. Although the type of racial discrimination experienced was not assessed in the 2008–9 NATSISS, other research suggests that racist talk is the most common experience of racism in Australia [24], including for Indigenous people [22]. It may be that Indigenous people have become inured to everyday experiences of racial discrimination such as racist talk, and therefore do not report it as a ‘problem’ for themselves, their family or close friends.

With respect to the settings in which racial discrimination was most commonly reported, findings from the 2008–9 NATSISS were somewhat similar to results from the Darwin Region Urban Indigenous Diabetes (DRUID) Study, in which the MIRE instrument (the basis for the module used in the NATSISS) was developed [13,25]. In the DRUID study, which involved a non-random local area Indigenous sample, racial discrimination was most commonly reported in employment and public settings, with service provision rather than legal settings as the third most common [25]. Males were more likely to report racial discrimination in legal and recreational settings across both studies (although the difference in relation to recreation was non-significant in the 2008–9 NATSISS). In the LEAD survey, racial discrimination was most commonly reported in shops, public settings, sports and work (as well as in educational settings) [22].

Most people spend considerable time in work settings and, to a lesser degree, public settings. These are settings in which there is relatively little control over shared space and social interactions with others (including discriminatory encounters). Previous research suggests that the over-representation of Indigenous Australians in the criminal justice system is associated with racial discrimination from police [26]. There is also evidence of racial discrimination from security personnel [27] who were included along with members of the legal profession within this setting. It is concerning that racial discrimination was so common in this setting despite most people having relatively little exposure to such situations in comparison to domestic, work or public spaces.

Several factors were significantly associated with reporting of racial discrimination in this study, including a broad range of demographic, socioeconomic and cultural factors. Previous studies have indicated that men report more experiences of racial discrimination than women [7,17,28,29], and this was found in some settings in the 2008–9 NATSISS. Current evidence is inconsistent with respect to variation in racial discrimination by age [7,17], perhaps due in part to curvilinear relationships as found among 2008–9 NATSISS respondents in this study. It is notable that both self-reported racial discrimination and reporting of discrimination as a stressor peaked in the centre of life’s prime (35–44 years). Perhaps this is the time of life when expectations of fair treatment are greatest and hence discrimination is most noticeable. Alternatively, people in their prime may be perceived as most threatening and hence most likely to be targets of discrimination, given a strong relationship between racism and threat/anxiety [30,31]. Further research is required to distinguish between these two possibilities.

Unemployment was strongly associated with reporting racial discrimination in work settings, but also in most other settings as well. Given that the unemployed are by definition not working, this discrimination is likely to operate primarily in seeking work, although the 12 month time frame for reporting may have included periods of employment (and therefore discrimination at work). More detailed analysis of the 2008–9 NATSISS undertaken by others indicates that racial discrimination in workplace settings leads to reduced engagement of Indigenous people in the labour market, as a way of avoiding experiences of racial discrimination [32].

Education was associated with increased reporting of racial discrimination. This is consistent with previous results from both the DRUID Study [25] and the LEAD surveys [22]. There are three main explanations for the association between education and increased reporting of discrimination. More educated Indigenous people: (1) may have higher expectations about how others should treat them, a difference in interpretation rather than exposure; (2) are more likely to work and socialise with non-Indigenous people and hence be exposed to inter-racial discrimination which is the more common, albeit not only, form of racial discrimination [22]; and (3) are more likely to be the targets of discrimination because they defy stereotypes of Indigenous people as ignorant and uneducated [33].

In contrast to education, home ownership was associated with significantly reduced reporting of racial discrimination. However, among those reporting any racial discrimination, home ownership was associated with increased odds of reporting discrimination as a stressor. Although Indigenous home ownership has increased slightly in recent years, it is still much less common than for the non-Indigenous population (29% versus 72% in 2008) [34], and this pattern of reporting may reflect both the benefits and the stresses of home ownership achieved against the odds.

Other markers of socioeconomic status, including household income and area-level disadvantage, were not consistently associated with reporting of racial discrimination. In the case of household income, this may have resulted at least in part from the relatively high proportion of respondents with missing data. Previous research, mainly involving African Americans, suggests that higher socioeconomic status (SES) is associated with increased reporting of racial discrimination [7,17,29,35-37]. However, SES has also been associated with lower reported racial discrimination both in general [23] and in specific situations, such as growing up in a middle-class or well-off family [37].

Remoteness was associated with lower self-reported racial discrimination. Given that the preponderance of racism appears to be perpetrated by non-Indigenous people [22], it may be protective for Indigenous Australians to live in communities where they are the majority (or at least a sizable proportion of the population) rather than a small minority. While the NATSISS CURF can not be used to examine this hypothesis directly in the Australian setting, recent evidence from New Zealand indicates that greater ethnic density is associated with reduced exposure to racism as well as better health for Māori after adjusting for area-level deprivation [38].

The association between markers of cultural affiliation and participation and reporting of racial discrimination is consistent with findings from other studies that stronger racial identity is related to increased reporting of racism [39]. This finding is likely due to a combination of: (1) increased awareness and perception of racism associated with greater investment in and salience of cultural or racial identity; and (2) increased likelihood of experiencing racial discrimination due to greater visibility as Indigenous through heightened cultural affiliation and/or participation [40] (although see [41] for alternative evidence). There have been mixed findings on the role of racial identity as a moderator between racial discrimination and negative outcomes [41], with racial identity found to exacerbate [42], buffer (among native American adolescents) [43] and be unrelated to [44] health and social outcomes.

Low levels of trust were also strongly associated with self-reported discrimination in the present study. Trust and racism can be related in various ways, including reduced levels of trust resulting from past experiences of racial discrimination [45,46].

Relatively few factors distinguished between those who reported racial discrimination and those who said they avoided situations due to past discrimination. Having a university degree, participating in cultural activities and having low levels of trust were associated with lower reported avoidance of situations due to past experiences, while living in a remote area was associated with greater reported avoidance of situations. Avoidance was most commonly reported for work, public and legal settings. Keeping in mind that the question about avoiding situations was only asked of people who *did not report any recent racial discrimination*, this question may be a reflection of people’s differing ability to *successfully* avoid settings and situations in which discrimination is likely to occur, rather than an indicator of whether or not people are *attempting* to avoid situations which have been problematic in the past. It is likely that many people tried to avoid situations in which they had experienced racial discrimination in the past but were unable to avoid it completely. In this case, they would be classified as having experienced racial discrimination, rather than as avoiding situations. It is likely that some settings and situations are easier to avoid than others, but any such avoidance, even if successful, is likely to incur personal, social, health and/or economic costs.

### Strengths and limitations

The main strengths of this study are the use of a national probability-based sample and a comprehensive module on racial discrimination experiences and stress. However, the type of racism experienced, such as racist attack, unfair treatment, exclusion, and racist talk [24] was not assessed in the 2008–9 NATSISS; this should be considered as a topic in further iterations of this survey.

One limitation of the present study is that estimated under-coverage in the 2008–9 NATSISS was relatively large compared with previous Indigenous surveys conducted by the Australian Bureau of Statistics [10]. If those who experienced more racism were less likely to participate in the 2008–9 NATSISS, then prevalence estimates could have been biased downwards. However, such under-coverage is unlikely to affect relationships between variables among 2008–9 NATSISS respondents as analysed in this paper. More generally, racial discrimination is difficult to accurately measure. An event caused by other factors may be considered discriminatory while some ‘objectively’ racist events may not be perceived as such by survey respondents. Vigilance bias is associated with increased (over-) reporting while minimisation bias is associated with decreased (under-) reporting of interpersonal racism. Research suggests that respondents are more likely to under-estimate personal experiences of racism [47-54] as well as experiences in society generally [55]. As such, the prevalence estimates reported here are likely to be a conservative estimate of the extent of racial discrimination experienced by Indigenous Australians. In any case, it is important to note that perceptions (even when inaccurate) are themselves important in driving health and social outcomes, and that relationships among variables are less likely to be affected by such biases than estimates of prevalence.

## Conclusion

The 2008–9 NATSISS provides the first detailed, national, population-based data on the patterns and correlates of racial discrimination as reported by Indigenous Australian adults. These data indicate that exposure to racial discrimination is common, especially in a few key settings, and that various socio-demographic and cultural factors are associated with different levels of reporting. Data such as these are critical to the development and evaluation of anti-racism policy, including efforts to reduce both the overall level of exposure to racism as well as the harms associated with such exposure. Although data from a single survey can be useful in highlighting areas of concern and identifying potential targets for intervention, it is critical to measure changes over time in order to monitor Australia’s progress as a nation in increasing equity and reducing exposure to racial discrimination.

## Endnotes

^a^ NAIDOC Week is an annual national celebration of Indigenous Australian culture, history and achievement [56].

^b^ This refers to Indigenous children of mixed racial/ethnic parentage who, between 1910 and 1970, were forcibly removed from their natural families by government authorities and placed in institutions or foster homes, with the intention of weakening their links to Indigenous society and strengthening their affiliation to White society [57].

## Competing interests

The authors developed the Measure of Indigenous Racism Experiences (MIRE) instrument on which the NATSISS racial discrimination module was based and provided advice to the ABS on the measurement of racism experiences in the Australian Indigenous population.

## Authors’ contributions

JC undertook all data analysis. JC and YCP contributed equally to conception and design, data interpretation, writing, revision and approval of the final manuscript. Both authors read and approved the final manuscript.

## Supplementary Material

Additional file 1: Table S7 Relative odds of self-reported racial discrimination in the last 12 months in public settings†, Indigenous Australians aged 15 years and over, 2008-09‡,§. Click here for file

Additional file 2: Table S8 Relative odds of self-reported racial discrimination in the last 12 months by the police, security people, lawyers or in a court of law, Indigenous Australians aged 15 years and over, 2008-09†,‡. Click here for file

Additional file 3: Table S9 Relative odds of self-reported racial discrimination in the last 12 months when applying for work or at work, Indigenous Australians aged 15-64 years, 2008-09†,‡. Click here for file
