# Supplementary material for: Patterns and correlates of self-reported racial discrimination among Australian Aboriginal and Torres Strait Islander adults, 2008–09: analysis of national survey data
Source: Int J Equity Health. 2013 Jul 1;12:47. doi: 10.1186/1475-9276-12-47 (PMC3703299; doi:10.1186/1475-9276-12-47)
Supplement: Additional file 1: Table S7 — Relative odds of self-reported racial discrimination in the last 12 months in public settings†, Indigenous Australians aged 15 years and over, 2008-09‡,§. [file 1475-9276-12-47-S1.pdf]

Additional File 1

Table 7. Relative odds of self-reported racial discrimination in the last 12 months in public settings†, Indigenous Australians aged 15 years and over, 2008-09.‡,§

	Model 1‖ OR (95% CI)	Model 2‖ OR (95% CI)	Model 3‖ OR (95% CI)	Model 4‖ OR (95% CI)
Married	0.7 (0.6-0.9)*	0.8 (0.6-1.0)	---	0.9 (0.6-1.1)
Remote area residence	0.8 (0.6-1.1)	0.8 (0.6-1.1)	---	0.5 (0.3-0.8)**
Highest qualification				
University degree	2.3 (1.3-4.0)**	2.8 (1.4-5.2)**	---	2.2 (1.2-4.1)*
Diploma/certificate	1.3 (0.9-1.8)	1.4 (1.0-2.1)	---	1.3 (0.9-1.9)
Year 12 only	0.8 (0.5-1.3)	0.9 (0.6-1.5)	---	0.9 (0.6-1.5)
Year 10/11 only	1.0	1.0	---	1.0
<Year 10 only	1.1 (0.8-1.6)	1.0 (0.7-1.5)	---	1.0 (0.7-1.5)
Labour force status				
Employed	1.0	1.0	---	1.0
Unemployed	1.8 (1.2-2.6)**	1.7 (1.2-2.6)**	---	1.5 (0.9-2.3)
Not in labour force	1.2 (0.9-1.6)	1.2 (0.9-1.7)	---	1.2 (0.8-1.7)
Home owned or being purchased	0.5 (0.4-0.7)***	0.4 (0.3-0.6)***	---	0.6 (0.4-0.8)**
Equivalised household income quintile				
1 (lowest)	1.0	1.0	---	1.0
2	0.8 (0.6-1.2)	1.0 (0.7-1.4)	---	1.0 (0.7-1.5)
3	0.9 (0.6-1.5)	1.1 (0.6-1.8)	---	1.3 (0.7-2.4)
4	0.7 (0.4-1.2)	0.9 (0.5-1.6)	---	1.1 (0.6-2.0)
5 (highest)	1.8 (0.7-4.7)	2.2 (0.8-6.0)	---	2.6 (0.9-7.5)

Not known/Not stated	0.9 (0.6-1.4)	1.1 (0.7-1.6)	---	1.2 (0.8-1.7)
SEIFA quintile				
1 (most disadvantaged)	1.0	1.0	---	1.0
2	1.3 (0.8-2.0)	1.3 (0.8-2.0)	---	1.5 (0.9-2.4)
3	0.7 (0.5-1.1)	0.8 (0.5-1.2)	---	0.9 (0.5-1.4)
4	1.5 (0.9-2.6)	1.7 (1.0-2.9)*	---	1.9 (1.1-3.4)*
5	1.0 (0.4-2.5)	1.1 (0.4-2.6)	---	1.1 (0.4-3.3)
Main language not English	0.9 (0.6-1.4)	---	0.5 (0.3-0.7)**	0.8 (0.5-1.4)
Household members all Indigenous	2.0 (1.5-2.7)***	---	1.5 (1.0-2.1)*	1.4 (0.9-2.0)
Identifies with clan, tribal, language group	3.4 (2.4-4.6)***	---	2.2 (1.5-3.2)***	2.1 (1.4-3.1)***
Identifies homelands	3.5 (2.5-5.0)***	---	1.9 (1.2-2.9)**	1.9 (1.2-2.9)**
Participated in cultural events, past 12 months	2.3 (1.6-3.3)***	---	1.3 (0.8-2.0)	1.3 (0.9-2.0)
Taken away from natural family	2.4 (1.7-3.5)***	---	2.0 (1.4-3.1)**	2.0 (1.3-3.0)**
% friends who are Indigenous				
Most or all	1.0	---	1.0	1.0
About half	1.1 (0.8-1.6)	---	1.2 (0.8-1.8)	1.0 (0.7-1.5)
Few	0.4 (0.3-0.5)***	---	0.6 (0.4-0.8)**	0.5 (0.3-0.7)***
Level of trust				
High	1.0	---	1.0	1.0

Medium	1.6 (1.1-2.1)**	---	1.5 (1.1-2.1)*	1.5 (1.1-2.0)*
Low	2.5 (1.9-3.4)***	---	2.7 (2.0-3.6)***	2.6 (2.0-3.5)***

† E.g. on the street, on public transport, or at shopping centres, parks, libraries, sporting events, concerts, restaurants, pubs or clubs [11].

‡ Source: Weighted data from the National Aboriginal and Torres Strait Islander Social Survey 2008-09 confidentialised unit record file (CURF) [11,12].

§ Includes only those with complete data on all variables of interest. Comparison group is those reporting no discrimination in any setting (total N=5,926).

‖ Model 1: Adjusted for age group and sex and the variable shown.
Model 2: Adjusted for age group, sex and the socio-demographic variables listed.
Model 3: Adjusted for age group, sex, and the cultural variables listed.
Model 4: Adjusted for age group, sex, and the socio-demographic and cultural variables listed.

* $p < 0.05$; ** $p < 0.01$; *** $p < 0.001$
