# Supplementary material for: Patterns and correlates of self-reported racial discrimination among Australian Aboriginal and Torres Strait Islander adults, 2008–09: analysis of national survey data
Source: Int J Equity Health. 2013 Jul 1;12:47. doi: 10.1186/1475-9276-12-47 (PMC3703299; doi:10.1186/1475-9276-12-47)
Supplement: Additional file 2: Table S8 — Relative odds of self-reported racial discrimination in the last 12 months by the police, security people, lawyers or in a court of law, Indigenous Australians aged 15 years and over, 2008-09†,‡. [file 1475-9276-12-47-S2.pdf]

Additional File 2

Table 8. Relative odds of self-reported racial discrimination in the last 12 months by the police, security people, lawyers or in a court of law, Indigenous Australians aged 15 years and over, 2008-09.†,‡

	Model 1§ OR (95% CI)	Model 2§ OR (95% CI)	Model 3§ OR (95% CI)	Model 4§ OR (95% CI)
Married	0.7 (0.5-0.9)**	0.8 (0.6-1.0)	---	0.9 (0.7-1.2)
Remote area residence	0.8 (0.6-1.0)	0.7 (0.5-0.9)*	---	0.4 (0.3-0.6)***
Highest qualification				
University degree	1.6 (0.8-3.2)	2.1 (1.0-4.5)*	---	1.8 (0.8-3.7)
Diploma/certificate	1.2 (0.9-1.8)	1.4 (0.9-2.0)	---	1.3 (0.9-2.0)
Year 12 only	0.7 (0.4-1.1)	0.8 (0.5-1.4)	---	0.9 (0.5-1.6)
Year 10/11 only	1.0	1.0	---	1.0
<Year 10 only	1.4 (1.0-2.0)	1.3 (0.9-1.8)	---	1.2 (0.8-1.9)
Labour force status				
Employed	1.0	1.0	---	1.0
Unemployed	2.3 (1.5-3.4)***	2.0 (1.4-2.9)***	---	1.8 (1.2-2.8)**
Not in labour force	1.4 (1.0-1.9)*	1.2 (0.9-1.6)	---	1.2 (0.8-1.6)
Home owned or being purchased	0.5 (0.3-0.6)***	0.4 (0.3-0.7)***	---	0.6 (0.4-0.9)*
Equivalised household income quintile				
1 (lowest)	1.0	1.0	---	1.0
2	0.7 (0.5-1.0)*	0.9 (0.6-1.3)	---	0.9 (0.6-1.4)
3	0.8 (0.5-1.3)	1.1 (0.7-1.8)	---	1.3 (0.8-2.1)
4	0.5 (0.2-0.9)*	0.7 (0.3-1.5)	---	0.9 (0.4-1.8)
5 (highest)	1.3 (0.4-4.0)	1.9 (0.6-6.5)	---	2.6 (0.6-11.1)

Not known/Not stated	0.9 (0.6-1.2)	1.1 (0.8-1.5)	---	1.1 (0.8-1.6)
SEIFA quintile				
1 (most disadvantaged)	1.0	1.0	---	1.0
2	1.2 (0.8-1.8)	1.2 (0.8-1.8)	---	1.5 (1.0-2.2)*
3	0.6 (0.4-0.9)*	0.6 (0.4-1.0)	---	0.8 (0.5-1.3)
4	1.1 (0.6-2.1)	1.3 (0.7-2.3)	---	1.7 (0.9-3.2)
5	0.6 (0.3-1.5)	0.8 (0.3-2.0)	---	0.9 (0.3-2.5)
Main language not English	0.8 (0.6-1.2)	---	0.4 (0.2-0.5)***	0.7 (0.4-1.0)
Household members all Indigenous	2.2 (1.6-2.9)***	---	1.4 (1.0-2.0)	1.2 (0.8-1.9)
Identifies with clan, tribal, language group	2.5 (1.8-3.5)***	---	1.7 (1.2-2.4)**	1.6 (1.1-2.3)*
Identifies homelands	2.5 (1.7-3.5)***	---	1.3 (0.9-2.0)	1.4 (0.9-2.1)
Participated in cultural events, past 12 months	2.7 (1.9-3.7)***	---	1.7 (1.1-2.6)*	1.9 (1.2-2.8)**
Taken away from natural family	3.0 (2.1-4.1)***	---	2.5 (1.7-3.6)***	2.2 (1.5-3.3)***
% friends who are Indigenous				
Most or all	1.0	---	1.0	1.0
About half	0.6 (0.4-0.9)*	---	0.6 (0.4-0.9)*	0.5 (0.3-0.7)**
Few	0.3 (0.2-0.4)***	---	0.4 (0.2-0.5)***	0.3 (0.2-0.4)***
Level of trust				
High	1.0	---	1.0	1.0

Medium	1.5 (1.1-2.0)*	---	1.4 (1.0-2.0)*	1.3 (0.9-1.9)
Low	2.6 (1.9-3.5)***	---	2.7 (2.0-3.6)***	2.6 (2.0-3.5)***

† Source: Weighted data from the National Aboriginal and Torres Strait Islander Social Survey 2008-09 confidentialised unit record file (CURF) [11,12].

‡ Includes only those with complete data on all variables of interest. Comparison group is those reporting no discrimination in any setting (total N=5,869).

§ Model 1: Adjusted for age group and sex and the variable shown.
Model 2: Adjusted for age group, sex and the socio-demographic variables listed.
Model 3: Adjusted for age group, sex, and the cultural variables listed.
Model 4: Adjusted for age group, sex, and the socio-demographic and cultural variables listed.

* $p < 0.05$; ** $p < 0.01$; *** $p < 0.001$
