# Supplementary material for: Patterns and correlates of self-reported racial discrimination among Australian Aboriginal and Torres Strait Islander adults, 2008–09: analysis of national survey data
Source: Int J Equity Health. 2013 Jul 1;12:47. doi: 10.1186/1475-9276-12-47 (PMC3703299; doi:10.1186/1475-9276-12-47)
Supplement: Additional file 3: Table S9 — Relative odds of self-reported racial discrimination in the last 12 months when applying for work or at work, Indigenous Australians aged 15-64 years, 2008-09†,‡. [file 1475-9276-12-47-S3.pdf]

Additional File 3

Table 9. Relative odds of self-reported racial discrimination in the last 12 months when applying for work or at work, Indigenous Australians aged 15-64 years, 2008-09.†,‡

	Model 1§ OR (95% CI)	Model 2§ OR (95% CI)	Model 3§ OR (95% CI)	Model 4§ OR (95% CI)
Married	0.8 (0.6-1.1)	0.8 (0.6-1.1)	---	0.8 (0.6-1.2)
Remote area residence	0.7 (0.5-0.9)**	0.7 (0.5-1.0)*	---	0.5 (0.3-0.7)**
Highest qualification				
University degree	2.7 (1.5-4.8)**	3.1 (1.6-5.9)**	---	2.6 (1.3-4.9)**
Diploma/certificate	1.3 (0.9-2.0)	1.3 (0.9-1.9)	---	1.2 (0.8-1.9)
Year 12 only	1.0 (0.6-1.6)	1.0 (0.6-1.7)	---	1.1 (0.7-1.8)
Year 10/11only	1.0	1.0	---	1.0
<Year 10 only	0.7 (0.4-1.0)	0.7 (0.5-1.1)	---	0.7 (0.4-1.1)
Labour force status				
Employed	1.0	1.0	---	1.0
Unemployed	3.0 (2.1-4.2)***	3.0 (2.0-4.4)***	---	2.7 (1.8-4.2)***
Not in labour force	0.5 (0.3-0.8)**	0.6 (0.4-0.9)*	---	0.6 (0.4-0.9)*
Home owned or being purchased	0.6 (0.5-0.9)**	0.5 (0.3-0.7)***	---	0.6 (0.4-1.0)*
Equivalised household income quintile				
1 (lowest)	1.0	1.0	---	1.0
2	0.9 (0.6-1.4)	0.9 (0.6-1.3)	---	0.9 (0.6-1.4)
3	1.3 (0.8-2.2)	1.3 (0.8-2.1)	---	1.5 (0.9-2.7)
4	0.9 (0.6-1.4)	0.8 (0.5-1.4)	---	0.9 (0.5-1.7)
5 (highest)	1.9 (0.9-4.0)	1.7 (0.7-4.0)	---	2.1 (0.9-5.1)

Not known/Not stated	1.0 (0.7-1.5)	1.1 (0.7-1.6)	---	1.1 (0.7-1.7)
SEIFA quintile				
1 (most disadvantaged)	1.0	1.0	---	1.0
2	1.4 (0.9-2.0)	1.3 (0.8-2.0)	---	1.4 (1.0-2.2)
3	0.8 (0.5-1.3)	0.8 (0.5-1.3)	---	0.8 (0.5-1.3)
4	1.4 (0.8-2.3)	1.4 (0.8-2.6)	---	1.6 (0.9-3.0)
5	1.6 (0.4-5.6)	1.4 (0.4-5.7)	---	1.6 (0.3-7.4)
Main language not English	0.7 (0.4-1.0)*	---	0.4 (0.2-0.6)***	0.7 (0.4-1.2)
Household members all Indigenous	1.6 (1.1-2.3)*	---	1.2 (0.8-1.9)	1.2 (0.7-2.0)
Identifies with clan, tribal, language group	2.4 (1.7-3.4)***	---	1.4 (1.0-2.1)	1.2 (0.8-1.8)
Identifies homelands	3.7 (2.4-5.8)***	---	2.5 (1.5-4.2)***	2.6 (1.5-4.4)**
Participated in cultural events, past 12 months	2.2 (1.5-3.1)***	---	1.4 (0.9-2.1)	1.3 (0.9-2.0)
Taken away from natural family	2.5 (1.7-3.8)***	---	2.1 (1.3-3.2)**	2.3 (1.4-3.7)**
% friends who are Indigenous				
Most or all	1.0	---	1.0	1.0
About half	1.2 (0.8-1.8)	---	1.1 (0.7-1.7)	0.8 (0.5-1.3)
Few	0.4 (0.3-0.6)***	---	0.5 (0.4-0.8)**	0.4 (0.2-0.6)***
Level of trust				
High	1.0	---	1.0	1.0

Medium	1.4 (0.9-2.0)	---	1.2 (0.8-1.9)	1.2 (0.8-1.9)
Low	2.0 (1.4-2.8)***	---	2.0 (1.4-2.8)***	1.9 (1.3-2.8)***

† Source: Weighted data from the National Aboriginal and Torres Strait Islander Social Survey 2008-09 confidentialised unit record file (CURF) [11,12].

‡ People aged 65 years and over have been excluded due to problems in estimation resulting from very low reporting in older age groups. Includes only those with complete data on all variables of interest. Comparison group is those aged 15-64 years reporting no discrimination in any setting (total N=5,286).

§ Model 1: Adjusted for age group and sex and the variable shown.
Model 2: Adjusted for age group, sex and the socio-demographic variables listed.
Model 3: Adjusted for age group, sex, and the cultural variables listed.
Model 4: Adjusted for age group, sex, and the socio-demographic and cultural variables listed.

* $p < 0.05$; ** $p < 0.01$; *** $p < 0.001$
